# Study of Profile of Mucormycosis During the Second Wave of COVID-19 in a Tertiary Care Hospital

**DOI:** 10.7759/cureus.21054

**Published:** 2022-01-09

**Authors:** Sangita Kamath, Manish Kumar, Nilanjan Sarkar, Tauheed Ahmed, Ashok Sunder

**Affiliations:** 1 Internal Medicine, Tata Main Hospital, Jamshedpur, IND; 2 Radiology, Tata Main Hospital, Jamshedpur, IND

**Keywords:** immunocompromised, mucor, covid-19, infections, fungal

## Abstract

Introduction and aim

Mucormycosis is a lethal opportunistic infection caused by filamentous fungi of the family Mucoraceae (black fungus). There has been a sudden increase in the incidence of these cases during the second wave of the COVID-19 pandemic due to the immunocompromised state caused by the disease and its treatment. Early diagnosis and appropriate medical management are essential to reduce disease morbidity and mortality. Through this study, we aim to study the clinical features, risk factors, laboratory investigations, and radiological findings of patients with mucormycosis as well as evaluate the clinical outcomes in each case.

Methods and materials

This was a prospective study that included only confirmed mucormycosis cases admitted in Tata Main Hospital (TMH) from April 2021 to July 2021. A case of mucormycosis was defined as the one in which clinical and radiological features were consistent with mucormycosis and fungus was demonstrated in the tissue by potassium hydroxide (KOH) mount/culture/histopathological examination (HPE). Data relating to epidemiology, risk factors, clinico-radiological features, and outcomes were analyzed and expressed as a percentage of total cases.

Results

Of the total 15 cases, three patients (33.3%) had active COVID-19 infection, eight (53.3%) were in the post-COVID-19 state, two (13.4%) had COVID-19 like illness and two (13.4%) patients did not have COVID-19 in the recent past. There was male predominance with the male to female ratio being 2.75:1. The commonest associated co-morbid condition was diabetes mellitus (13 patients, 86.7%). Amongst the myriad manifestations, periorbital swelling was the commonest symptom (11 patients, 73.3%). Among neurological manifestations, involvement of cranial nerves was found in nine (60%) patients with the third cranial nerve being the most commonly affected nerve (eight patients, 53.3%). Cavernous sinus thrombosis (CST) was found in one (6.7%) patient. Diagnostic nasal endoscopy (DNE) revealed eschar at various sites in 13 patients (86.7%). Central retinal artery occlusion (CRAO) was found bilaterally in one patient (6.7%) while two patients (13.3%) had CRAO on the left. Radiologically, the most commonly involved sinuses were maxillary and ethmoidal (eight patients, 53.3%). Bilateral sinus involvement was more common (46.7%) than unilateral sinus involvement. The average length of stay (LOS) was 17.5±7.8 days. The overall mortality was 40%. Five (33.3%) patients developed secondary bacterial infections. All patients received medical therapy with intravenous amphotericin B. In addition, seven (46.7%) patients underwent functional endoscopic sinus surgery (FESS) with debridement of which, five (71.4%) patients survived and made a good recovery. One patient (6.7%) with pulmonary mucormycosis underwent lobectomy.

Conclusion

New-onset headache, black nasal discharge, periorbital swelling, retro-orbital pain, visual diminution, restriction of eye movements should prompt an immediate search for mucormycosis especially in the background of history of diabetes mellitus in patient with recent or current COVID-19 disease. Radio-imaging with computerized tomography and magnetic resonance imaging are complementary to clinical evaluation in assessing the disease extent and diagnosis of complications. Prompt diagnosis is essential due to the angio-invasive nature of the mucor and requires aggressive anti-fungal therapy and debridement of the devitalized tissue.

## Introduction

Fungal infections once considered rare, have reemerged during the second wave of COVID-19 infection. The course of COVID-19 infection or post-COVID-19 state was often complicated by secondary fungal infections like mucormycosis and aspergillosis. While the prevalence of mucormycosis varied from 0.005 to 1.7 per million population worldwide, it was almost 80 times higher (0.14 per 1000) in India compared to that in the developed countries as per the data for the year 2019-2020 [1]. India alone has contributed to 71% of the total global cases. The disease, thus, emerged as a new imminent threat even as the pandemic continued to be a reality.

In India alone, about 14,872 cases of mucormycosis were reported as of May 28, 2021. Gujarat recorded the maximum number of cases, with 3,726 infections in active and recovered COVID-19 patients, followed by Maharashtra [2]. Other states like Rajasthan, Andhra Pradesh, Karnataka, Uttarakhand, and Delhi also showed a gradual increase in the number. The death toll due to the disease was 4,332 cases by July 2021 as per the data presented in the Rajya Sabha. In addition, a rising number of cases of aspergillus and candida infection also were reported.

Mucormycosis is an angioinvasive disease that occurs in patients with compromised immune function. It is caused by filamentous mold fungi of the genus Rhizopus, Mucor, and Rhizomucor, of the order- Mucorales and class- Zygomycetes [3]. Rhizopus oryzae is the most common type, which accounts for nearly 60% of mucormycosis cases in humans. It is also responsible for most of the rhino-orbital-cerebral (ROCM) forms of the disease [4]. The disease is transmitted by the inhaled fungal spores which are ubiquitously present in the decaying organic matter of the soil and or by inoculation of the spores into disrupted mucosa or abraded skin. Irrespective of the route of invasion, the hyphae cause invasion of blood vessels, resulting in tissue infarction and necrosis [5]. Microscopically, the hyphae are broad, aseptate, multinucleate (coenocytic), and demonstrate zygospores. They characteristically branch at right angles.

As this is still an emerging disease, the diagnosis is often made late, especially in those presenting with uncommon symptoms. Timely diagnosis and medical management are essential to save the life and reduce morbidity. Patients who were treated within six days have a survival rate of 76 to 81%, while a delay in treatment of more than 12 days reduces the rate to 36 to 42% [6, 7]. 

We, therefore studied this cluster of fifteen cases admitted to Tata Main Hospital during the peak of the second COVID-19 wave with an aim to study the clinical features, risk factors, laboratory investigations, and radiological findings of patients with mucormycosis and evaluate the clinical outcomes in each case.

## Materials and methods

This was a prospective study conducted in Tata Main Hospital which is a 960-bedded tertiary care hospital in Jamshedpur, Jharkhand. The study population constituted admitted patients with the following inclusion criteria during the study period from April 2021 to July 2021 (three months). This corresponded with the peak of the second wave of COVID-19 pandemic. The study was given clearance by the Institutional Ethics Committee (IEC). Written informed consent was taken from the participants of the study.

Inclusion criteria 

Confirmed cases of mucormycosis with or without other fungal infections as proven by microscopic examination of the aspirate or histopathology of tissue specimens.

Exclusion criteria

Cases where the microbiological diagnosis could not be confirmed.

Data collection techniques and tools

The case files of these patients were prospectively analyzed for symptoms and findings of clinical examination of the ear, nose, and throat (ENT), eye, and central nervous system to assess the extent of the disease. Underlying comorbidities, disease course, findings of diagnostic nasal endoscopy (DNE) in case of involvement of nose or sinuses, biochemical and hematological investigations, findings of the radiological examination, fungal elements isolated, treatment instituted, and disease outcomes were noted.

The development of mucormycosis in a subject with a present or recent diagnosis of COVID-19 [real time -polymerase chain reaction (RT-PCR) positive for severe acute respiratory syndrome coronavirus 2 (SARS-CoV-2)] was defined as COVID associated mucormycosis (CAM). Cases were defined as early CAM when mucormycosis was diagnosed within seven days and late CAM when they occurred eight days or more after COVID-19 diagnosis [8]. The time interval between the appearance of the first COVID-19 symptom and that of mucormycosis was noted.

Investigations included complete blood picture, blood sugars, liver function tests (serum bilirubin, alanine aminotransferase (ALT), aspartate aminotransferase (AST), alkaline phosphatase (ALP), serum proteins (albumin and globulin) prothrombin time, international normalized ratio (INR), c- reactive protein and renal function tests (blood urea and serum creatinine). These were done using automated biochemical analyzers, on the day of admission and repeated every 72 hours till discharge or death. Three-tesla magnetic resonance imaging (MRI), plain [T1, T2/T2 fluid attenuation inversion recovery sequence (FLAIR), T2 fat suppression (FS) and with contrast (gadolinium) of paranasal sinuses (PNS), orbits, and brain was done in all patients with suspected rhino-orbital-cerebral involvement unless there was contra-indication. Cases were classified into three groups depending on the extent of involvement based on the classification suggested by Rupa et al. [9] as follows.

Stage 1: Involvement of nose and paranasal sinuses only

Stage 2: Additional spread to orbit/palate/oral cavity

Stage 3: Extensive disease (extension into the intracranial region, periorbital region, or pterygopalatine fossa)

DNE was done in case of clinical involvement of nose, sinuses, or eye and in post-operative functional endoscopic sinus surgery (FESS) cases and it was repeated every 48 to 72 hours to assess the progression of the disease or response to therapy. Samples were taken for culture and swabs for KOH preparation. Mucormycosis was diagnosed by finding broad aseptate, ribbon-like hyphae with right-angled branching while that of aspergillosis was made by demonstrating septate hyphae in the histological examination of biopsy samples obtained during debridement or from potassium hydroxide (KOH) wet mount of the nasal swab obtained during DNE or other samples.

Sample processing

The biopsy or aspirated material was collected in sterile containers of normal saline and 10% formalin. The microscopic examination of wet-mount specimen in 10-20% potassium hydroxide (KOH) was done to identify the fungus. Tissue sections were stained with hematoxylin and eosin (H&E) stain or lactophenol cotton blue (LPCB) stain for histopathological examination. For culture, the tissue sample was cut into small pieces and inoculated into a Sabouraud dextrose agar (SDA) medium with antibiotics.

Treatment protocol

All patients were examined by a multidisciplinary team comprising of a neurologist, ophthalmologist, otorhinolaryngologist, physician, microbiologist, and pathologist. They were managed according to protocolized institutional treatment for mucormycosis which included liposomal amphotericin B (LAmB) given intravenously in the dose of 5 milligrams (mg)/kg body weight infused over three hours in 5% dextrose solution. This was started as soon as the diagnosis of mucormycosis was established and continued for three weeks while monitoring for nephrotoxicity. The patient was adequately hydrated with 500 ml of 0.9% normal saline before and after the infusion. In case liposomal amphotericin B was not available, 1.0-1.5 mg/kg/day of amphotericin B deoxycholate (D-AmB) was used. Parenteral amphotericin B was followed by oral posaconazole 300 mg twice a day on day one followed by 300 mg once daily for three to six months depending upon the response to therapy. Oral posaconazole was also started if the patient did not tolerate or developed side effects to parenteral amphotericin B. Treatment was also instituted to correct any underlying metabolic derangement. FESS with surgical debridement was done to remove the necrotic tissue by otorhinolaryngologist where indicated. Also, exenteration of the affected eye was planned in non-salvageable cases. Those with aspergillosis were treated with a combination of LAmB and posaconazole.

Clinical outcomes

Clinical outcomes studied included length of stay (LOS), complications, response to treatment, and mortality.

Statistical analysis

Continuous variables were expressed as mean ± SD. For categorical variables, the percentage of patients in each category was calculated. Odds ratio (OR) with 95% confidence limits (CL) and relative risk (RR) were used to calculate the association between the risk factors and the severity of mucormycosis. 

## Results

**Figure 1:**
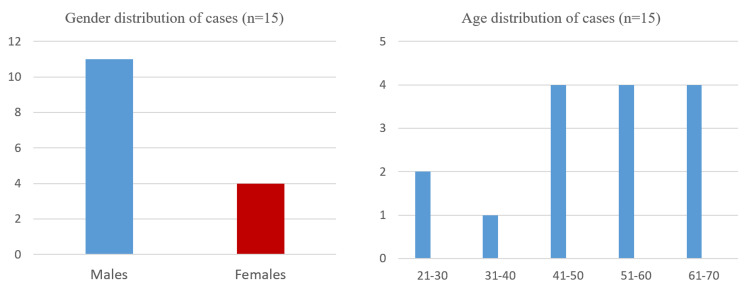
Gender and age distribution of cases

Of the total of 15 patients with mucormycosis, 11 (73.3%) were male and four (26.7%) were female patients. The male to female ratio was 2.75:1. Their age ranged from 27 to 70 years with mean (±SD) being 50.8 (±12.96) years. While 13 (86.7%) patients were in the 41 to 70 years age group, only 2 (13.3%) presented before the fourth decade of life. The age and sex distribution of cases are shown in Figure 1. All patients were from urban areas. Five patients (33.3%) were from lower socio-economic class while 10 (66.6%) were from upper socio-economic class.

Three patients (20%) had developed mucormycosis while they were positive for SARS-CoV2 infection, eight patients (68.5%) were in the post-COVID-19 state, two (13.3%) had COVID-19-like illness, and two (13.3%) did not have a history of COVID-19 in the recent past but had other comorbidities like diabetes mellitus (DM), hypertension, and chronic kidney disease (CKD). All three patients of COVID-19 had severe disease with computed tomography severity score (CTSS) being more than 20/25 and had received 125 mg methylprednisolone intravenously once daily for 10 days along with remdesivir for five days, cefepime 1 gram twice daily, supplemental oxygen therapy in the form of oxygen with reservoir bag and high flow nasal oxygen (HFNO). None had required mechanical ventilation. One of the three patients had early CAM. He developed symptoms of mucor on day three of diagnosis of SARS-CoV2 infection (early CAM), while the other two patients had late CAM.

Amongst the patients in post-COVID19 state, the average duration of post-COVID-19 state before the diagnosis of mucormycosis was 20.6 days (range 12 to 45 days). Four of the eight patients had moderate COVID-19 and had received dexamethasone 6 mg once daily for 10 days and remdesivir for five days, while one patient had mild disease and had symptomatic treatment at home. Data was not available for three patients.

The commonest co-morbid condition observed in our study was DM. It was found in 13 (86.7%) of our patients. All except one had uncontrolled diabetes with average random blood sugar being more than 350 mg/dl. The average HbA1c levels of five patients were 11.9%. Diabetic ketoacidosis (DKA) was found in two (13.3%) patients during admission. Additionally, four (26.7%) patients had essential hypertension, two (13.3%) had CKD and hypothyroidism each, and one (6.7%) was a renal transplant recipient. No comorbidities were found in two (13.3%) patients.

**Figure 2:**
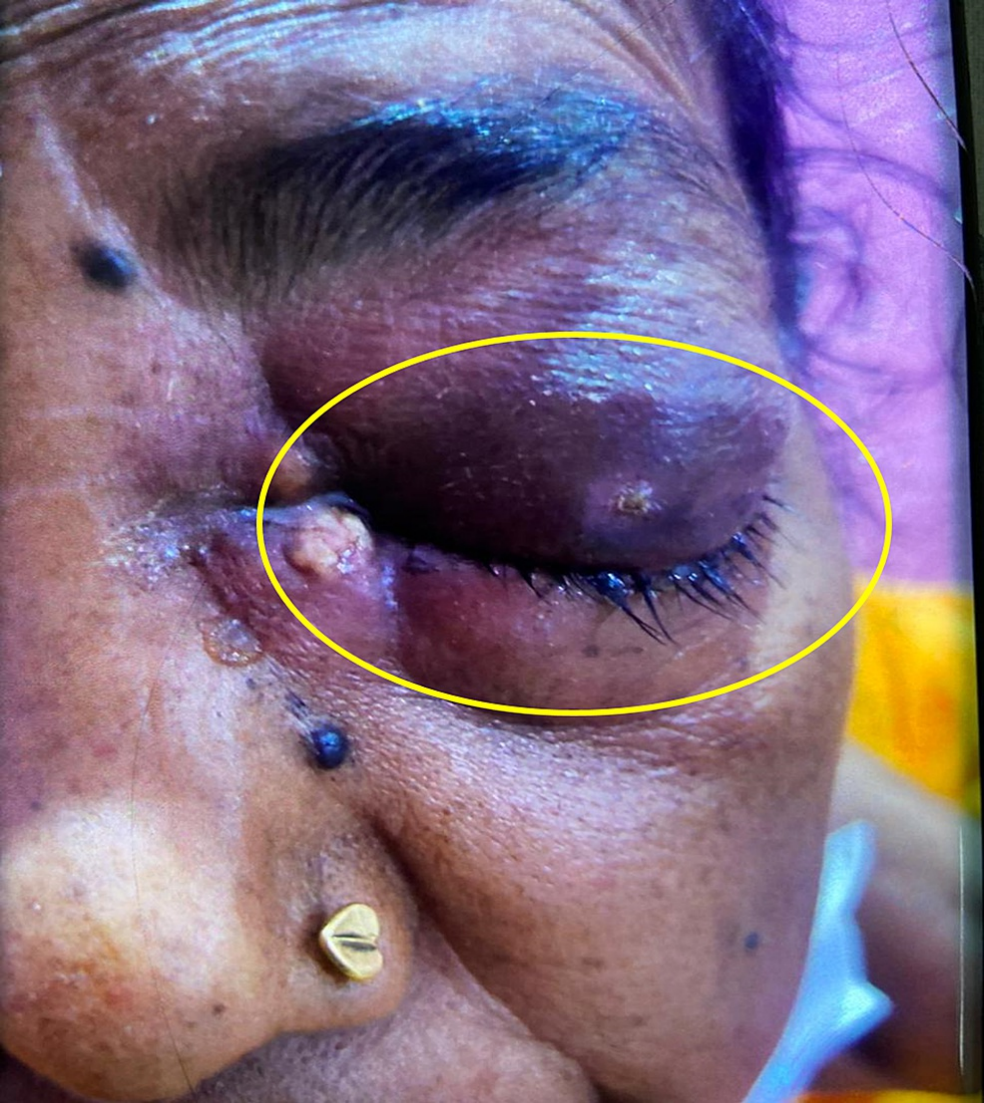
Left periorbital swelling in a patient of mucormycosis

**Figure 3:**
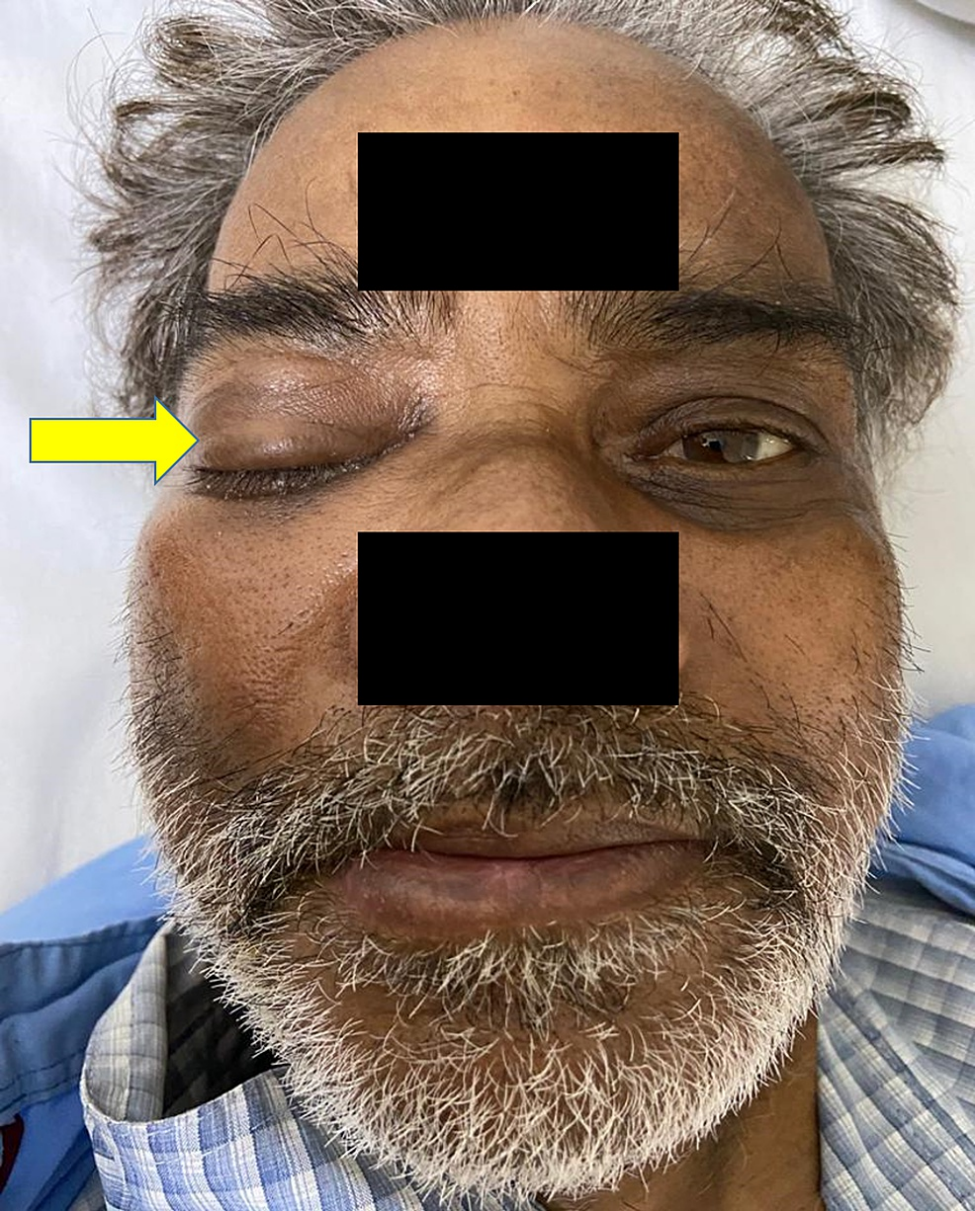
Ptosis of right eye in a patient with mucormycosis. He also had complete external ophthalmoplegia.

The diverse clinical manifestations (Figures 2, 3) in descending order were as summarized in Table 1. 

**Table 1:** Profile of symptoms of patients of mucormycosis (n=15)

Clinical symptoms	Number of cases(%) (n=15)
Periorbital swelling	11 (73.3)
Facial pain	10 (66.7)
Retro-orbital pain	10 (66.7)
Visual impairment	9 (60)
Drooping of upper eyelid (ptosis)	8 (53.3)
Cheek swelling	7 (46.7)
Upper jaw tooth pain	6 (40)
Unilateral nasal discharge	5 (33.3)
Fever	5 (33.3)
Proptosis (bulging of the eyeball)	4 (26.7)
Headache	3 (20)
Altered sensorium	3 (20)
Cellulitis of cheek	1 (6.7)
Double vision for far objects	1 (6.7)
Facial deviation	1 (6.7)
Dysarthria	1 (6.7)
Tinnitus	1 (6.7)

Periorbital swelling was the commonest symptom noted in 11 (73.3%) of the patients (Figure 2) while ptosis was observed in eight (53.3%) patients (Figure 3).

The average duration of fever was 6.6 days. Nasal discharge was bloody in one (6.7%) patient while two (13.3%) had a black-colored discharge. Involvement of cranial nerves was found in nine (60%) patients. The third cranial nerve was the most commonly affected nerve (eight patients, 53.3%). Seven patients had complete third nerve involvement, while one patient had partial involvement. Other cranial nerves involved were fourth (five patients, 33.3%), sixth (six patients, 46.7%), seventh (two patients,13.3%), maxillary and mandibular divisions of fifth (two cases,13.3%), and eighth (one patient, 6.7%). A maximum of five cranial nerves (third, fifth, sixth, seventh, and eighth) were affected in one patient. Six (40%) patients did not have evidence of cranial nerve involvement. Objective clinical signs related to various cranial nerves involvement were as depicted in Table 2. Amongst the various clinical signs, abnormalities of the extraocular movement were the commonest (eight patients, 53.3%), of which isolated lateral rectus paresis was seen in one subject (6.7%).

**Table 2:** Clinical signs of subjects with mucormycosis (n=15)

Clinical signs	Number of cases (%) (n=15)
Restriction of extraocular movements (External opthalmoplegia)	8(53.3)
Fixed and dilated pupils (Internal ophthalmoplegia)	7(46.7)
Facial numbness	2(13.3)
Facial muscles weakness with deviation	1(6.7)
Loss of corneal reflex	1(6.7)
New-onset hearing difficulty	1 (6.7)

**Figure 4:**
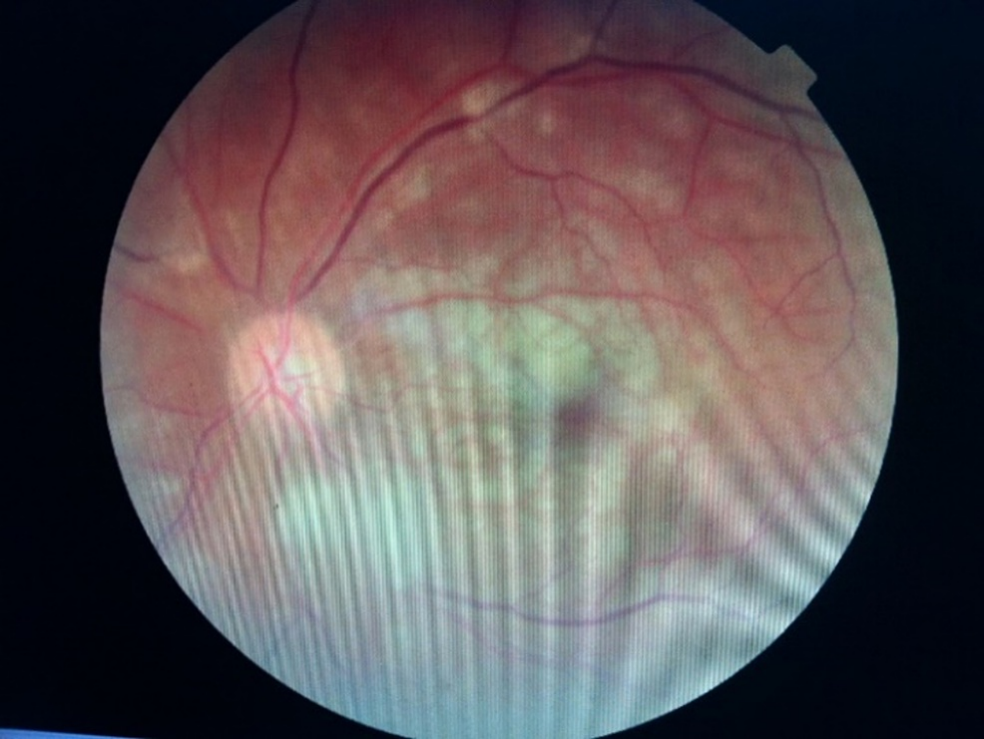
CRAO in left eye in a patient with mucormycosis

In addition to the signs related to the involvement of cranial nerves, sinus tenderness was found in 10 (66.7%) patients. One patient had ear involvement with mucopurulent discharge. Examination of the ear revealed congested and edematous tympanic membrane with pulsatile discharge in the posterior-superior quadrant. Fundus examination revealed evidence of central retinal artery occlusion (CRAO) in both eyes in one patient (6.7%) while two patients (13.3%) had CRAO on the left (Figure 4). Evidence of cavernous sinus thrombosis (CST) by magnetic resonance imaging (MRI) was found in one (6.7%) patient. 

**Figure 5:**
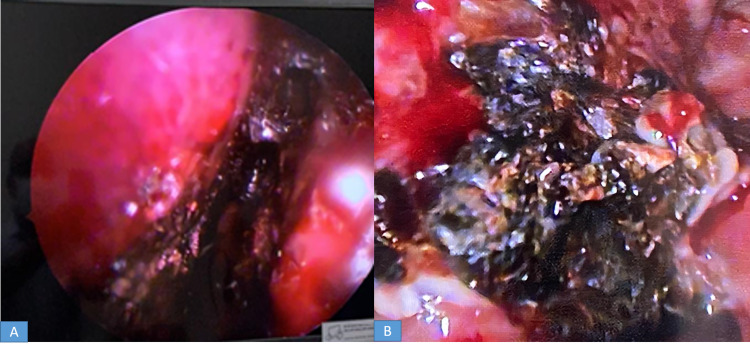
A and B - Diagnostic nasal endoscopy showing blackish discoloration of the nasal mucosa by mucor

DNE revealed eschar (blackish necrotic tissue) over nasal mucosa bilaterally (Figures 5A, 5B) in three (20%) patients, over the left middle turbinate in three (20%) patients, covering right middle turbinate in two (13.3%) patients, over the spheno-ethmoidal recess in three (20%) patients, over the hard palate in three (20%) patients, over the superior turbinate in one (6.7%) patient, and over the inferior turbinate in one (6.7%) patient. Eschar over the nasopharynx was found in one patient while mucopurulent discharge through the middle meatus was found in two (13.3%) patients and in one (6.7%) patient, anterior and posterior ethmoidal sinuses, sphenoidal sinus and right maxillary sinus were filled with pus. DNE was normal in two patients. Further, deviated nasal septum, a well-known predisposing factor for fungal rhinosinusitis, was found in three (20%) of our patients.

The average leucocyte count ± standard deviation (SD) of these patients on admission was 20,093.3 ± 10,494.4 cu/mm while average serum ferritin levels were 1153.2 ± 646.4 microgram/liter. Radiologically the most common involved sinuses were maxillary and ethmoidal (eight, 53.3%). Involvement of all the sinuses was seen in six cases (40%). The least common affected were frontal and sphenoidal sinuses. They were involved as a part of pansinusitis or when at-least three sinuses were affected. Their isolated involvement was not seen. Sinus involvement on both sides was more common (46.7%) than unilateral sinus involvement. Profile of sinus involvement in mucormycosis was as described in Table 3. Three (20%) patients could not be subjected to radiological investigations related to paranasal sinuses for various reasons.

**Table 3:** Sinuses involved in mucormycosis (n=15)

Sinuses involved	Number (%)
Pansinusitis	6 (40)
Isolated maxillary	4 (26.7)
Isolated frontal	0
Isolated ethmoid	0
Isolated sphenoid	0
Ethmoidal + maxillary + sphenoidal	1 (6.7)
Frontal + maxillary + ethmoidal	1 (6.7)

**Figure 6:**
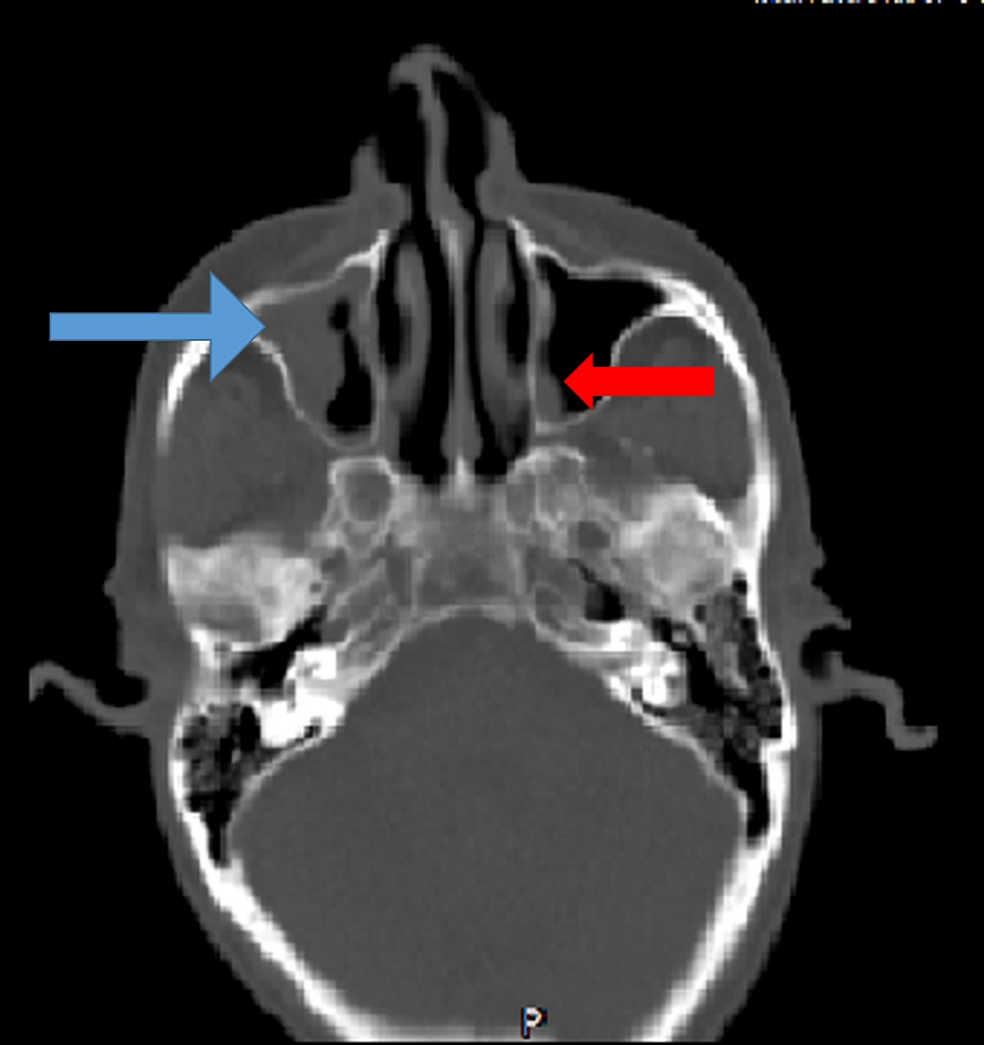
Axial CT head showing polypoidal mucosal thickening of right maxillary sinus (blue arrow) and focal thickening of left maxillary sinus (red arrow)

**Figure 7:**
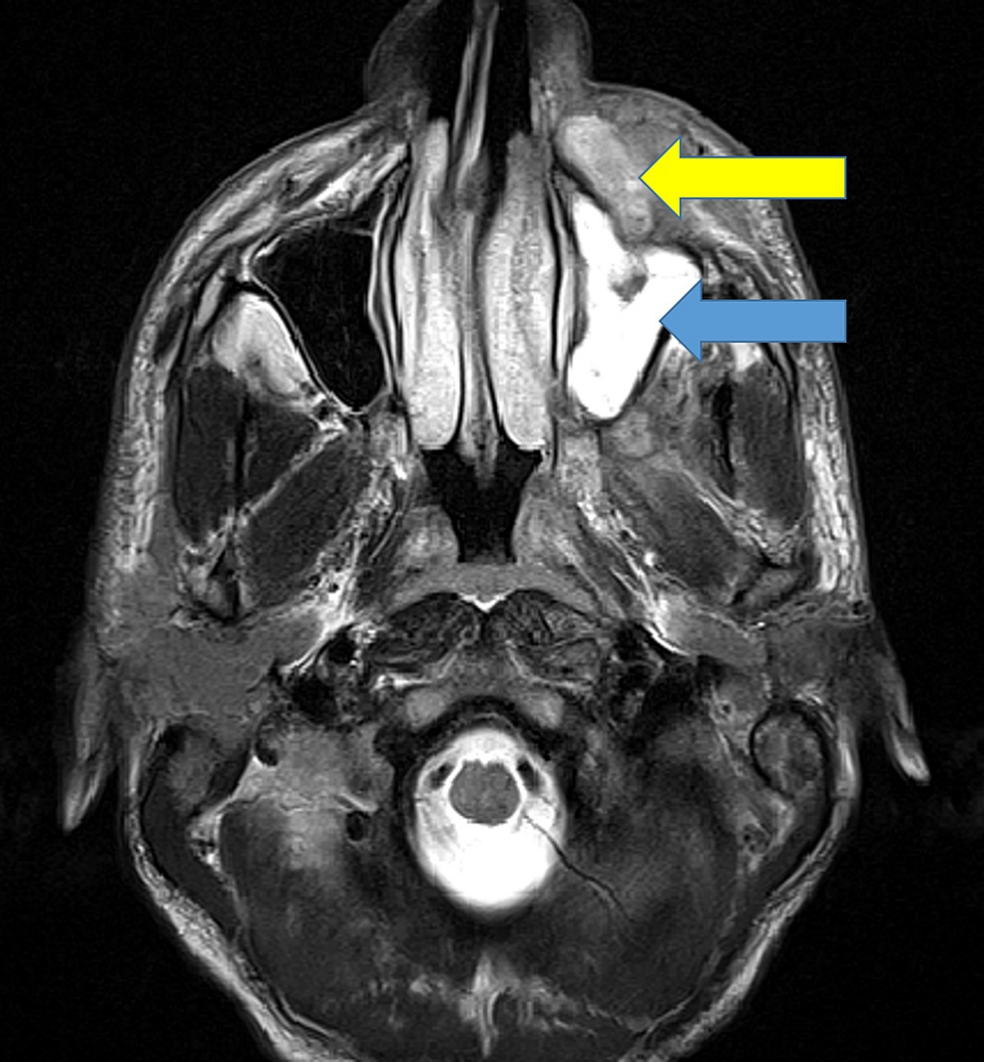
MRI-T2 weighted axial section showing mucosal enhancement of left maxillary sinus (blue arrow) with left cheek abscess (yellow arrow) anterior to the maxillary sinus T: transverse relaxation time

**Figure 8:**
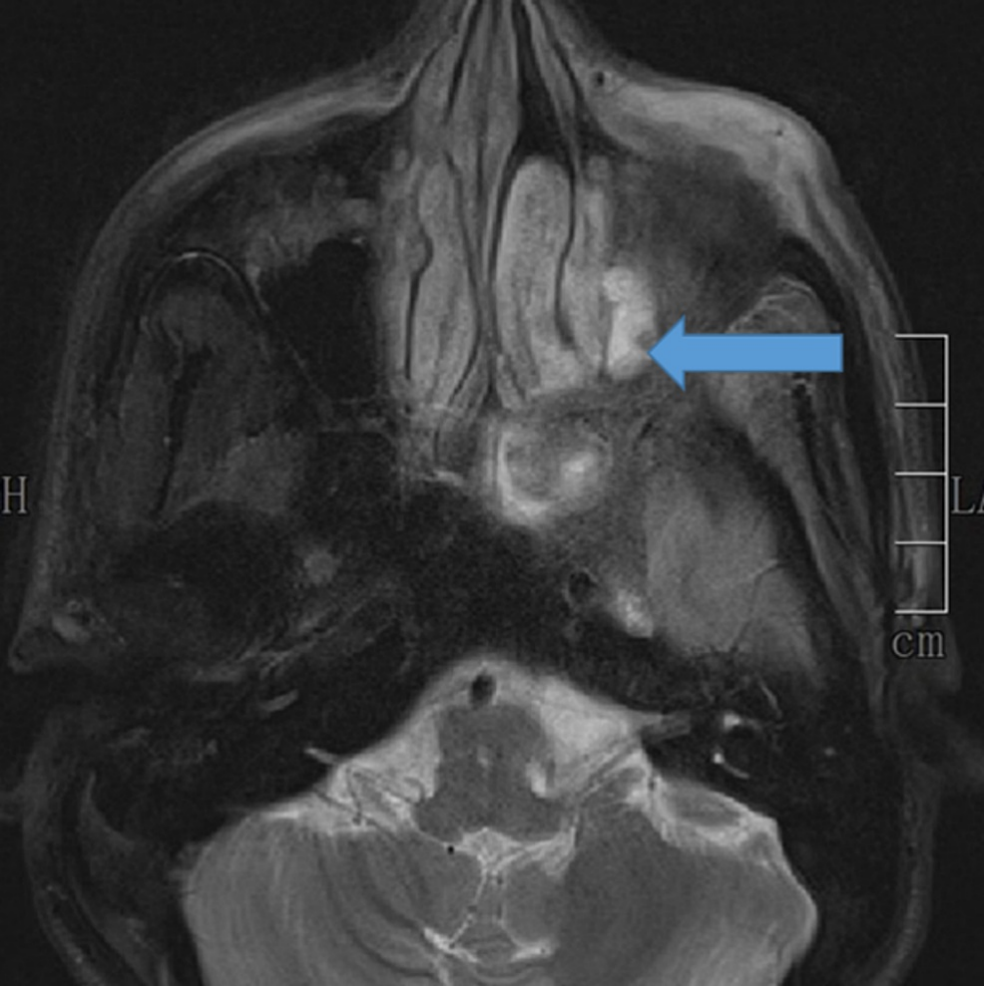
MRI- Fat suppression (FS) image showing extra-conal left orbital involvement

**Figure 9:**
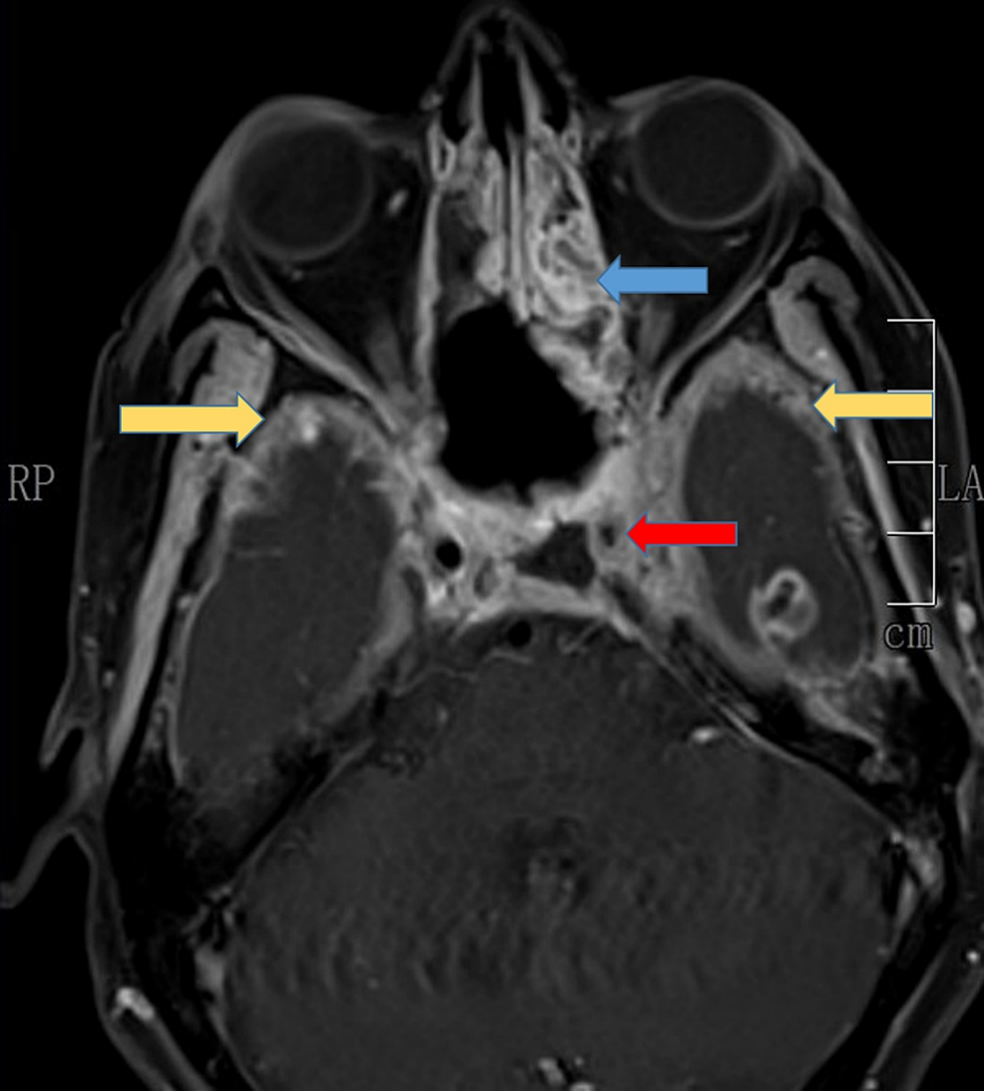
MRI brain, axial view showing partial thrombus in the cavernous portion of ICA (red arrow), left ethmoidal sinusitis (blue arrow), and meningeal enhancement over temporal lobes bilaterally (yellow arrow) ICA: internal carotid artery

The MRI findings of paranasal sinuses (PNS), orbits, and brains of the patients were tabulated in Table 4, Figures 6, 7, 8, 9. Clinical symptoms and signs were confined to one side but features of bilaterally radiological involvement were seen in four (25.8%) patients.

**Table 4:** Radiological (MRI) findings and microbiological diagnosis in admitted patients (n=15) PNS: paranasal sinuses, FNAC: fine needle aspiration cytology, KOH: potassium hydroxide, ICA: Internal carotid artery, SOV: Superior ophthalmic vein

Case	MRI PNS	MRI orbits	MRI Brain	Tissue sent/Microbiological diagnosis
1	Mucosal thickening with enhancement and fluid collection in right maxillary, ethmoidal and sphenoidal sinuses with air-fluid levels	Orbital extra-ocular muscles enhancement (medial and superior recti and inferior oblique)	Normal	Sinus tissue biopsy-mucormycosis
2	Not done	Not done	Not done	FNAC of left supraclavicular mass
3	Pansinusitis, sparing frontal sinuses, air-fluid levels in maxillary antrum on both sides	Right orbital apex enhancement	Not done	Nasal tissue biopsy-mucormycosis + Aspergillosis
4	Mucosal thickening with enhancement of both maxillary sinuses	Normal	Normal	Nasal tissue biopsy-mucormycosis
5	Mucosal thickening with enhancement of all sinuses – pansinusitis, bilateral	Right orbital apex involvement	Infratemporal fossa and cavernous sinus enhancement on right	Nasal tissue biopsy-mucormycosis
6	Pansinusitis - Mucosal thickening with enhancement of all sinuses on right	Right orbital apex involvement	Normal	Nasal tissue biopsy-mucormycosis
7	Mucosal thickening of right maxillary sinus with fat stranding	Right orbital apex involvement with proptosis	Normal	Nasal tissue biopsy-mucormycosis
8	Left maxillary sinus mucosal thickening	Left retro-orbital mass penetrating left basi-temporal region, infiltrating into (breaching) the lamina papyracea	Left frontal and parietal cerebritis with left cavernous sinus enhancement	Nasal tissue biopsy-mucormycosis
9	Mucosal thickening of right maxillary sinus with fat stranding in the retro and pre-maxillary space and heterogeneity in right masticator space	Orbital soft tissue involvement with mild proptosis on right	Normal	Sinus tissue biopsy-mucormycosis + aspergillosis
10	Normal paranasal sinuses	Involvement of the lateral and inferior orbital walls on right	Normal	KOH wet mount of nasal swab- mucormycosis
11	Pansinusitis-mucosal thickening with bilateral obstruction of osteomeatal complex	Normal	Normal	Sinus tissue biopsy-mucormycosis
12	Right frontal, maxillary, bilateral ethmoidal and sphenoidal sinuses involved	Enhancement of soft tissue in superior portion of the right orbit	Middle cranial fossa parenchymal enhancement with right ICA* and bilateral cavernous sinuses flow void suggestive of thrombosis and bilateral SOV^#^ dilated	Sinus tissue biopsy-mucormycosis
13	Not done as ear, nose and throat were not involved clinically			Broncho-alveolar lavage (BAL) specimen
14	Not done as ear, nose and throat were not involved clinically			Parapharyngeal abscess – mucormycosis + aspergillosis
15	Pansinusitis, soft tissue thickening of the left maxillary sinus and medial part of masticator space, stranding of pre-maxillary fat	Enhancement of left orbital apex	Few lacunar infarcts in deep white matter of the brain	Maxillary antrum tissue biopsy-mucormycosi

**Figure 10:**
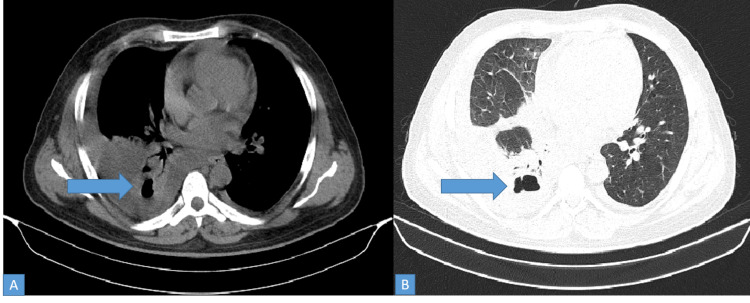
CT thorax showing collapse, consolidation with cavitation in right lower lobe (A-bone and B-lung windows)

CECT thorax in one patient with pulmonary mucormycosis who presented with lower chest and upper abdominal pain, revealed collapse, consolidation with the cavity in right lower lobe with mild pleural effusion (Figures 10A, 10B). He subsequently developed non-resolving cavitating pneumonia.

**Figure 11:**
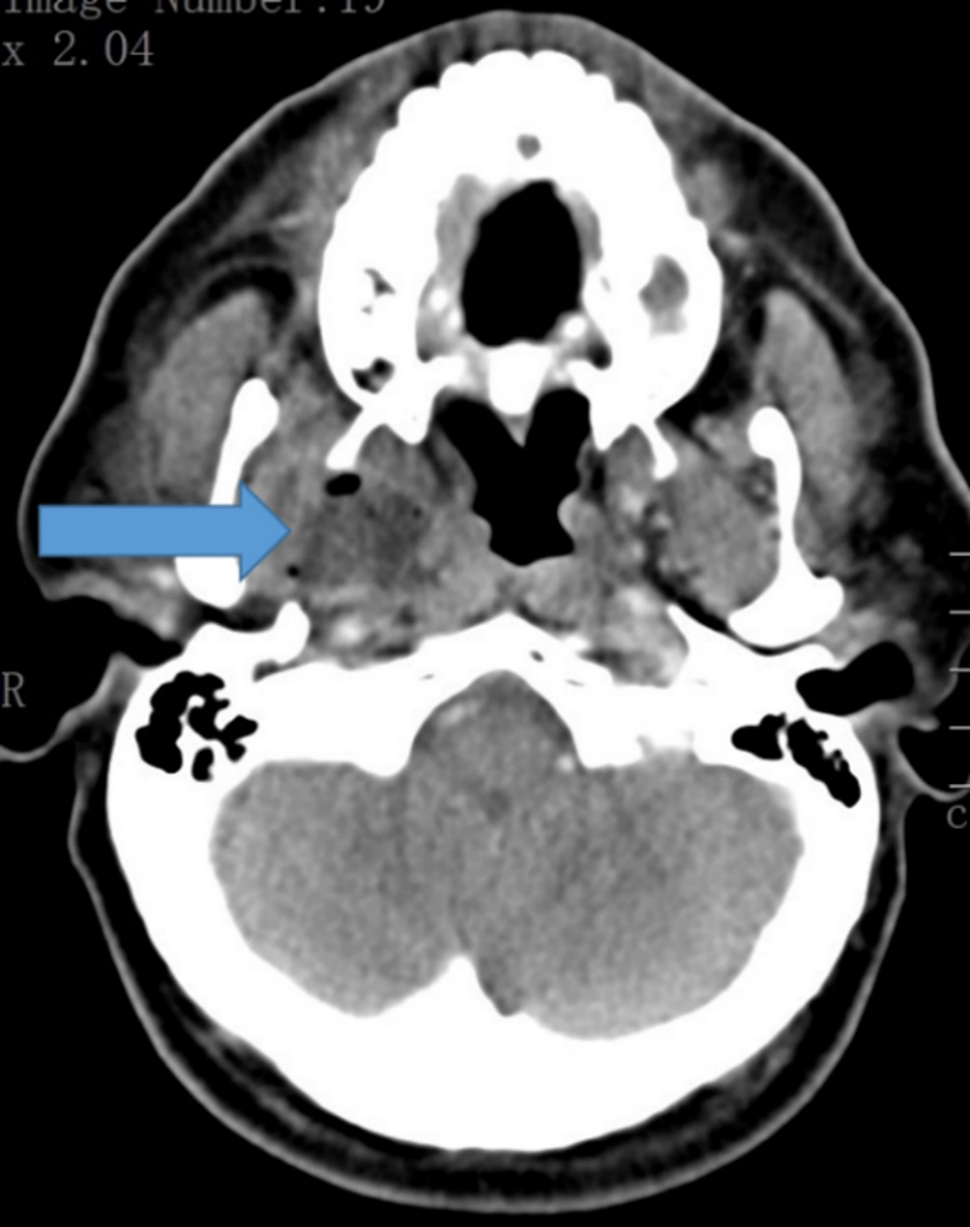
CT scan of head - axial view showing right parapharyngeal abscess

KOH wet mount of bronchoalveolar lavage (BAL) obtained during bronchoscopy revealed mucor (patient number 13). Yet another patient developed a right para pharyngeal abscess and had presented with odynophagia (Figure 11). KOH mount of the aspirated pus showed mucor and aspergillus (patient number 14).

**Figure 12:**
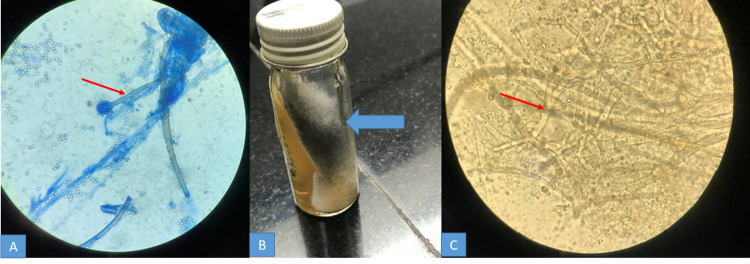
A - Photomicrograph showing mucor from the nasal biopsy taken from a patient. B - Black growth of mucor in SDA medium and C - Demonstration of the fungal hyphae SDA: Sabouraud's dextrose agar

Microbiological diagnosis: KOH wet mount preparation demonstrated mucor in 5 (33.3%) cases, while the culture of the biopsied material/pus grew the fungus in 10 (66.6%) cases (Figures 12A, 12B, 12C). 

**Figure 13:**
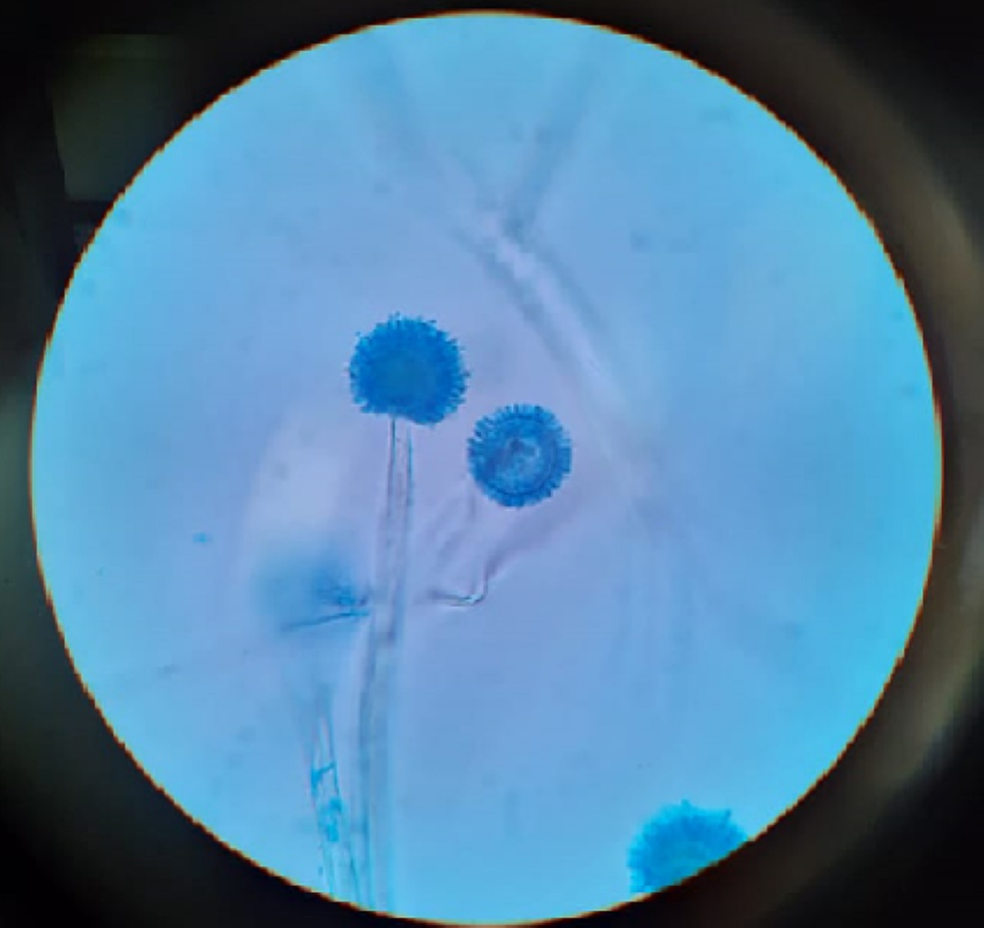
Photomicrograph showing aspergillus from the nasal biopsy taken from patient with mucormycosis

There was evidence of angio-invasion in eight (53.3%) cases. In 3(20%) cases there was mixed infection with mucor and aspergillus (Figure 13).

Clinical outcomes

The average length of stay (LOS) was 17.5±7.8 days. Six patients expired giving rise to mortality of 40%. Four (26.7%) patients were discharged against medical advice (DAMA). Five (33.3%) patients developed secondary bacterial infections. While two patients developed empyema thoracis on the right, one patient developed serratia marcescens septicemia with subsequent septic shock and multi-organ dysfunction syndrome (MODS). Culture of the pus from the nasal cavity grew Klebsiella pneumoniae in one (6.7%) patient. Yet another patient (6.7%) developed bloodstream infection with Pseudomonas aeuroginosa. One (6.7%) patient with a right parapharyngeal abscess developed osteomyelitis of the right mandibular ramus.

All patients received protocolized medical therapy with intravenous amphotericin B as described above. In addition, seven (46.7%) patients underwent functional endoscopic sinus surgery (FESS) with debridement. Five out of the seven (71.4%) patients who underwent FESS survived and made a good recovery. Four transcutaneous retro-orbital injections of amphotericin B (3.5 mg/ml) [TRAMB] were given to a patient who did not give consent for exenteration. One patient (6.7%) with pulmonary mucormycosis underwent lobectomy at a higher center. Clinical diagnosis, staging, treatment, and the outcomes were as shown in Table 5.

**Table 5:** Outcomes of the patients with mucormycosis (n=15) FESS: Functional endoscopic sinus surgery, TRAMB: Transcutaneous retro-orbital Amphotericin B (3.5 mg/ml – 4 injections), DAMA: discharge against medical advice.

Case number	Clinical Diagnosis	Stage of the disease	Surgery	Outcome Survived	Outcome/ Expired
1	Rhino-sino-orbital mucormycosis	Stage 2	FESS + debridement	-	Expired
2	Left supraclavicular mass	Not done	Not done	DAMA	-
3	Rhino-sino-orbital mucormycosis with aspergillosis	Stage 2	Not done	-	Expired before surgery
4	Rhino-sino-orbital mucormycosis	Stage 2	Not done	-	Expired before surgery
5	Rhino-sino-orbital-cerebral mucormycosis	Stage 3	FESS + debridement	Survived	-
6	Rhino-sino-orbital mucormycosis	Stage 2	FESS + debridement	-	Expired
7	Rhino-orbital-cerebral mucormycosis	Stage 3	Not done	-	Expired before surgery
8	Rhino-sino-orbital-cerebral mucormycosis	Stage 3	FESS + Denker’s medial maxillectomy + radical frontal, ethmoid and sphenoidectomy + TRAMB.	-	Expired
9	Rhino-sino-orbital mucormycosis with aspergillosis	Stage 2	FESS + debridement	Survived	-
10	Rhino-orbital mucormycosis	Stage 1	Exenteration planned	DAMA	
11	Sino-orbital mucormycosis	Stage 1	FESS + debridement	Survived	-
12	Rhino-sino-orbital mucormycosis	Stage 3	FESS + debridement	Survived (DAMA)	-
13	Pulmonary mucormycosis	Not done	Medical treatment + lobectomy of right lower lobe	Survived	
14	Parapharyngeal abscess (Mucormycosis with aspergillosis)	Not involved	Incision and drainage of abscess + saucerization and curettage of right ramus of the mandible	Survived	-
15	Rhino-sino-orbital- cerebral mucormycosis	Stage 3	FESS + left Caldwell Luc operation	Survived	-

Predictors of mortality

History of uncontrolled diabetes mellitus (DM), COVID 19 infection, severe COVID 19 (presence of pneumonia), steroid use, delay in initiating surgical therapy was apparently more in the expired group than in the survived group. Odds ratio (OR), 95% confidence interval (CI), and relative risk (RR) were as shown in Table 6.

**Table 6:** Predictors of severity of mucormycosis (n=15)

Risk factors	Total numbers survived (n=9)	Total numbers expired (n=6)	Odds Ratio	95%CI (confidence interval)	Relative risk (RR)
Uncontrolled DM	7 (77.8%)	6 (100%)	1.29	0.3 - 5.8	1.15
History of COVID-19 infection	7 (77.8%)	5 (83.3%)	1.07	0.2 - 5.0	1.04
Presence of COVID-19 pneumonia	4 (44.4%)	6 (100%)	2.25	0.4 - 11.5	1.5
Preceding steroid use	8 (88.9%)	6 (100%)	1.13	0.3 - 4.9	1.07
Delay in initiating surgical treatment	2 (22.2%)	5 (83.3%)	3.75	0.5 - 26.1	1.72

## Discussion

The second wave of COVID-19 was associated with an alarming increase in the rate of fungal infections throughout the world. More than 20 different fungal species have been identified in hospitalized COVID-19 patients. Common agents implicated have been Aspergillus fumigatus, Candida albicans, and mucormycosis [5]. In the Indian context, the rise in the incidence of mucormycosis has occurred at an unprecedented rate. Fungal co-infections were not unexpected in the current SARS-CoV-2 outbreak, as similar observations were made during previous outbreaks of other coronaviruses such as the severe acute respiratory syndrome-coronavirus (SARS-CoV) and the middle east respiratory syndrome coronavirus (MERS-CoV).

Mucormycosis is an angio-invasive fungal infection caused by saprophytic fungi of the order Mucorales and class Zygomycetes. It carries a high mortality in immunocompromised patients. Mucormycosis was ﬁrst described in 1876 by Fürbinger in Germany. He reported a patient who died of cancer, whose right lung showed a hemorrhagic infarct with fungal hyphae and sporangia [1]. The exact incidence is not known as there are few population-based studies. However, the incidence is rising over years with the increase in the incidence of diabetes, HIV infection, and malignancies globally. Out of the 119 clinically suspected cases, Chakrabarti et al. in north India reported 50 cases of fungal rhinosinusitis over two years period [10] while Anushuya G et al. reported 84 cases of the total 738 cases in a study from south India over a seven-year period [11]. Singh AK et al. reported 82 cases (81.2%) from India, nine (8.9%) from the United States, and three (3.1%) from Iran in a systematic review of 95 cases of mucormycosis in patients with COVID-19 [12].

Risk factors

Various factors have been proposed for the increase of mucormycosis infection in COVID-19 patients. These include hyperglycemia due to uncontrolled diabetes mellitus (DM) and the excessive use of corticosteroids for the treatment of hypoxemia in COVID-19. In a 2019 meta-analysis of 851 cases of mucormycosis by Jeong W et al., the presence of DM was found to be an independent risk factor for rhino-orbital-cerebral mucormycosis (odds ratio (OR) 2.49; 95% CI 1.77-3.54; p < 0.001) [13]. The same was observed in our study as well, with diabetes mellitus being the commonest co-morbid condition seen in 13 (86.7%) of our patients. The average HbA1c levels of five patients were 11.9%. Diabetic ketoacidosis (DKA) was found in two (13.3%) patients during admission. Similar findings were seen in a systematic review of 101 cases of mucormycosis in COVID-19 patients by Singh AK et al. [12]. Pre-existing diabetes mellitus (DM) was observed in 80% of his cases while diabetic ketoacidosis (DKA) during admission for mucormycosis was found in 14.9%.

Hyperglycemia causes glycosylation of transferrin and ferritin which reduces their iron-binding capacity, thus increasing free iron levels. Raised interleukin-6 levels in COVID-19 further increase free iron level through increased production of ferritin. In addition, there is upregulation of the expression of glucose regulator protein 78 (GRP-78) on endothelium cells and fungal ligand spore coat homolog (CotH) protein, which facilitates angio-invasion of the fungus, hematogenous dissemination, and tissue necrosis [12,14].

The use of long-term corticosteroids has traditionally been associated with a high risk of opportunistic fungal infections. Corticosteroids cause impairment of the function of several immune cells, such as polymorphonuclear leukocytes, T lymphocytes, monocytes, and macrophages. In the European Confederation of Medical Mycology study, 46% of the patients had received steroids within a month preceding the diagnosis of mucormycosis [15]. This was also evident in our study where seven of the 15 (46%) patients had received steroids in the form of methylprednisolone or dexamethasone. In a systematic review of 101 mucormycosis cases in COVID-19, the use of corticosteroid for the treatment of COVID-19 was present in 76.3% of cases [13]. A prospective study conducted in Wales showed that the use of corticosteroids in high-doses significantly increased the odds of COVID-19 patients developing aspergillosis (14.1%) [16]. Similarly, in Brazil, Riche et al. observed a 10-fold rise in candidemia in critically ill COVID-19 patients receiving high-dose steroids [17].

Prolonged stay in the intensive care unit (ICU), use of invasive ventilation, decreased phagocytic activity of white blood cells (WBC) caused by SARS-CoV-2 infection coupled with several pre-existing co-morbidities further compound the risk of fungal infection. The use of prophylactic fluconazole or voriconazole in these patients has also been linked with a rise in cases of mucormycosis. Acidosis and chronic iron therapy might have contributed to the progression of zygomycosis in two of our patients who had chronic kidney disease [18].

The use of interleukin 6 inhibitors like tocilizumab for treating patients with severe COVID-19 is also associated with increased risk, though none of our patients in the study had received it. It causes immune deregulation by interfering with both innate and adaptive immune responses through various mechanisms, thus increasing the patient’s susceptibility to invasive fungal infections.

In our study, the average duration of post-COVID-19 state before the diagnosis of mucormycosis was 20.6 days. This was significantly longer than in a previous study by Selarka et al. [19] where this duration was 12.1 days.

Clinical features and complications

Mucormycosis is classified as rhino-cerebral, pulmonary, cutaneous, gastrointestinal, or disseminated, depending on the organ involved, the most common form being rhino-cerebral (39%). This variant is further divided into subtypes based on which tissues are affected- rhino-nasal, rhino-orbital, or rhino-orbito-cerebral (ROCM). ROCM is the commonest variety seen worldwide [20].

The fungus is usually found as a commensal in the nasal mucosa. Fungal spores enter via inhalation or by the ingestion of contaminated food and subsequently enter the paranasal sinuses. Affected individuals initially develop symptoms of acute sinusitis like fever, nasal congestion, purulent nasal discharge, and headache. Contiguous spread to adjacent structures like orbit may occur through the nasolacrimal duct or through the medial wall of the orbit, resulting in various clinical symptoms like facial pain, chemosis, vision loss, protrusion of eyeball, limitation of ocular movements, periorbital or cheek swelling. Ophthalmoplegia occurs due to infection of the extra-ocular muscles and orbital space or when the third, fourth and sixth cranial nerves are involved either in the orbit or in the cavernous sinus. Lower motor neuron type seventh cranial nerve paresis or paralysis and hypoesthesia of the face (due to involvement of mandibular nerve) may also occur [21].

In our study, many of these clinical features were observed with periorbital swelling being the most common clinical manifestation, seen in 73% of patients. Cranial nerve involvement was seen in 9 (60%) patients, with the third cranial nerve being most commonly involved. In a review of 208 cases of mucormycosis by Yohai et al, [22] ophthalmoplegia was observed in 67% of cases, vision impairment in 65%, proptosis in 64%, periorbital edema in 43%, and periorbital pain in 11% in the descending order. Also, in the same study, 22% of patients had facial paralysis and 20% had facial hypoesthesia. In a study by Therakathu J et al., involving 43 patients of mucormycosis, 11 patients (25%) had various cranial nerve palsy while six patients had multiple cranial nerve palsies at presentation [23]. In our study, diplopia (double vision) was seen in one (6.7%) patient, though ophthalmoplegia was observed in eight (53.3%) cases. This could possibly be due to complete external ophthalmoplegia or associated ptosis, which results in a poor perception of double vision. This was similar to that reported by Dubey S et al. in their study on mucormycosis from Eastern India [24]. While unilateral complete ophthalmoplegia was observed in seven patients, one had bilateral ophthalmoplegia.

Central retinal artery occlusion (CRAO) is reportedly a rare manifestation of ROCM and has an incidence of 16%-20%. It is caused by direct infiltration of the central retinal artery (CRA) by angio-invasion of the fungus from the orbit resulting in necrotizing vasculitis. Bawankar P et al. reported a case of unilateral CRAO in a diabetic patient of mucormycosis who presented with a sudden loss of vision [6]. One patient in our study had bilateral CRAO with bilateral cavernous sinus thrombosis, which is hitherto not described in the literature.

Diagnosis

The diagnosis of mucormycosis was made by finding broad, aseptate, ribbon-like hyphae with right-angled branching in the histological examination of samples obtained from DNE or debridement. Imaging with computed tomography (CT) and/or magnetic resonance imaging (MRI) of the orbit, brain, and/or paranasal sinuses aids in the diagnosis and helps in assessing the extent of involvement from mucormycosis. In our study, on imaging, the most commonly involved sinuses were maxillary and ethmoidal (8, 53.3%). Involvement of all the sinuses was seen in six cases (40%). This was similar to a study by Sharma et al. [25] where ethmoids were the most commonly involved sinuses followed by maxillary sinus. Ethmoid sinus was the commonest paranasal sinus involved (37, 86%) in the study by Therakathu J et al. from South India [23]. In their study, unilateral sinus involvement was more common (79.1%) than bilateral sinus involvement (20.9%) as in our study where bilateral involvement was more common (46.7%). Meningeal involvement by imaging was seen in one (6.7%) patient.

Imaging helps in localizing the disease, detecting the disease extent, identiﬁcation of complications like thrombosis, and is invaluable for planning surgery [12]. It is very sensitive in picking up the initial stage of the disease, even before symptoms have appeared, as in four (26.7%) of our cases. Extra-sinus extension of the disease in the form of fat stranding in the pre-maxillary area, orbital fat stranding, and altered fat in pterygopalatine fossa aids in the early diagnosis of invasive fungal infection on imaging, in the backdrop of appropriate clinical setting [22].

Treatment

Treatment involves a multidisciplinary team approach. The mainstay of treatment is antifungals and surgical debridement of the affected tissues. The survival rate of mucormycosis patients has increased from 6% to around 60% with the availability of intravenous amphotericin B [26-28]. Surgical exploration and debridement help to limit the spread of infection and allow better penetration of intravenous drugs into the infected tissues. Seven (46.7%) of our patients underwent functional endoscopic sinus surgery (FESS) with debridement. Five out of the seven (71.4%) patients who underwent FESS survived and made a good recovery.

Mortality

Overall mortality in our study was 40%. Similar figures were seen in a cluster of 10 patients with sino-orbital mucormycosis with concurrent COVID-19 by Sarkar S et al. [29]. A recent systematic review of mucormycosis cases in India and worldwide by Jeong W et al. reported mortality of 30.7% [13]. Another study by White et al. involving 135 COVID-19 patients in Wales, also reported significantly higher mortality in patients with invasive fungal infections (53% vs 31%) than in patients without coexisting fungal infection [15]. The high mortality associated with mucormycosis is attributed to the rapidity of its dissemination. A delay of 12 hours in the diagnosis could prove fatal, which is why 50% of cases of mucormycosis have been diagnosed only in the post-mortem autopsy. Intracranial involvement in mucormycosis increases the fatality rate to as high as 90%. Cerebral involvement was seen in three (20%) of our patients of which two died.

The presence of uncontrolled DM, COVID-19 infection, severe COVID-19 pneumonia, steroid use, delay in initiating surgical therapy were apparently associated with a higher relative risk of severe mucormycosis and increased mortality. However, as the number of cases was small, the result was not statistically significant.

## Conclusions

Males, from the lower socio-economic group, were commonly affected. SARS-CoV2 infection (current or recent past), use of a high dose of corticosteroids, and a high prevalence of DM were the important risk factors involved in mucormycosis. Hence, judicious use of steroids along with tight glycemic control is essential to keep mucormycosis in check. While the diagnosis remains challenging, a high index of suspicion on the part of clinicians is needed, for early detection and aggressive management to decrease the morbidity and mortality of this dreadful disease.

The disease manifests with a myriad of clinical symptoms and signs among which periorbital swelling, diminution of vision, ptosis, external ophthalmoplegia, and facial pain are common. The disease often starts unilaterally, and later becomes bilateral with delay in treatment. Third, fourth, and sixth cranial nerves are commonly involved. Simple bedside tests like pupillary reaction, ocular, sinus tenderness, and palatal examination should be a part of routine physical evaluation of a COVID-19 patient. Neuroimaging is needed for localization of disease, to know its extent, and for early diagnosis of the clinically asymptomatic lesion. Direct examination, culture, and or histopathology are essential to confirm the diagnosis. When diagnosed, rapid initiation of antifungal therapy along with surgical debridement of devitalized tissue where appropriate, may help to improve the survival of these patients.
